# Organ preservation: current limitations and optimization approaches

**DOI:** 10.3389/fmed.2025.1566080

**Published:** 2025-03-26

**Authors:** Qiulin Ran, Jiayi Zhang, Jisheng Zhong, Ji Lin, Shuai Zhang, Guang Li, Bin You

**Affiliations:** ^1^Department of Cardiovascular Surgery, Beijing Institute of Heart Lung and Blood Vessel Diseases, Beijing Anzhen Hospital, Capital Medical University, Beijing, China; ^2^Translational Medicine Center, Beijing Chest Hospital, Capital Medical University, Beijing, China

**Keywords:** organ preservation, transplantation, static cold storage, machine perfusion, graft quality, organ repair

## Abstract

Despite the annual rise in patients with end-stage diseases necessitating organ transplantation, the scarcity of high-quality grafts constrains the further development of transplantation. The primary causes of the graft shortage are the scarcity of standard criteria donors, unsatisfactory organ preservation strategies, and mismatching issues. Organ preservation strategies are intimately related to pre-transplant graft viability and the incidence of adverse clinical outcomes. Static cold storage (SCS) is the current standard practice of organ preservation, characterized by its cost-effectiveness, ease of transport, and excellent clinical outcomes. However, cold-induced injury during static cold preservation, toxicity of organ preservation solution components, and post-transplantation reperfusion injury could further exacerbate graft damage. Long-term *ex vivo* dynamic machine perfusion (MP) preserves grafts in a near-physiological condition, evaluates graft viability, and cures damage to grafts, hence enhancing the usage and survival rates of marginal organs. With the increased use of extended criteria donors (ECD) and advancements in machine perfusion technology, static cold storage is being gradually replaced by machine perfusion. This review encapsulates the latest developments in cryopreservation, subzero non-freezing storage, static cold storage, and machine perfusion. The emphasis is on the injury mechanisms linked to static cold storage and optimization strategies, which may serve as references for the optimization of machine perfusion techniques.

## 1 Introduction

Donation after brain death (DBD), living donation, and extended criteria donors (ECD) constitute the primary sources of donor grafts. Despite the annual rise in patients with end-stage diseases necessitating organ transplantation worldwide, the total number of DBD and living organ donors remains mostly unchanged (1). The scarcity of high-quality donor organs has constrained the development of organ transplantation, making the enhancement of utilization and survival rates of ECD a pressing concern.

In recent decades, the development and application of various organ preservation solutions have established static cold storage (SCS) as the standard preservation practice for donated organs. This organ preservation technique is secure, simple, and easily transportable, while *ex vivo* dynamic machine perfusion (MP) was previously overlooked. Current SCS techniques are well established. However, SCS techniques and some components in organ preservation solutions may be hazardous (2, 3). Numerous current research studies reported the addition of diverse protective agents into existing organ preservation solutions, with the minimization or substitution of harmful constituents, to enhance the protective effects of organ preservation solutions. The literature mostly concerns a modified variant of the histidine-tryptophan-ketoglutarate (HTK) solution, known as the HTK-N solution. Preclinical evidence indicates that the HTK-N solution outperforms the HTK solution in organ preservation. The effectiveness and safety of HTK-N solution have been confirmed by a pilot randomized controlled clinical phase II trial in living donor transplantation of human kidney preservation, and the outcomes of a prospective, randomized, single-blind, multicenter, phase III study on kidney, liver, and pancreatic transplantation are currently unavailable (4, 5). Despite the fact that existing organ preservation solutions are being gradually improved, the absence of donor organ viability assessment, tissue damage from prolonged hypothermia, limited cold storage duration, and the ischemia–reperfusion injury (IRI) that inevitably occurs during transplantation have constrained the clinical application of ECD grafts (6–8). Contemporary technologies of cryopreservation and subzero non-freezing preservation remain significantly underdeveloped. This has led to a resurgence of interest in MP preservation.

*Ex vivo* dynamic MP facilitates the evaluation of graft viability and functional repair, hence alleviating IRI and reducing the risk of primary non-function and delayed graft function (DGF) (1, 9). Despite the high cost and technical complexity of MP preservation for transplants, along with the risk of organ waste in the event of preservation failure, this technology creates the conditions for the successful clinical application of ECD grafts. This review describes several preservation strategies, including cryopreservation, subzero non-freezing preservation, SCS, and MP, to enhance the reader’s comprehensive understanding of the diverse preservation alternatives for grafts. Furthermore, we emphasize the constraints of SCS and its related damage mechanisms, which may offer insights into the optimization of MP approaches.

## 2 Cryopreservation protocols

For decades, cryopreservation solutions have been widely utilized to freeze cells in liquid nitrogen or at −80°C refrigerators. To further diminish the toxicity of cryopreservation solutions and the possibility of pathogenic bacterial contamination, an increasing number of cryopreservation solutions are devoid of serum and dimethyl sulfoxide. While cell cryopreservation technology is secure and well-established, research on whole-organ cryopreservation remains in its nascent stages. The cryopreservation of kidneys and hearts has been extensively explored; nevertheless, organ function cannot be reinstated following cryopreservation at temperatures below −45°C (10). The primary causes of cryopreservation failure are the cytotoxic effects of cryoprotective agents (CPAs) and the formation of sharp ice crystals, which result in tissue matrix fragmentation and endothelial damage, leading to microvascular dysfunction, significant metabolic disturbances, and acute immune rejection (11, 12). Recent innovations in the composition of cryopreservation solutions and freezing and rewarming technologies have significantly enhanced the potential for the preservation of human whole organs.

Hydrogel encapsulation provides a non-toxic alternative to conventional cryopreservation (13, 14). Alginate is a non-toxic, biocompatible polymer, and alginate encapsulation is being used for both cryopreservation and non-cryopreservation of higher plants, animals, and human cells (15, 16). The thicknesses of 3% alginate or 5% gelatin-methacryloyl hydrogel encapsulation optimally preserved mouse testicular tissues by inhibiting ice crystal formation, minimizing basement membrane contraction, improving cell morphology, and augmenting mitochondrial activity (14). Vitrification is the rapid cooling of an organ to a stable, ice-free, glassy state, preventing solid ice injury to tissues; however, vitrification necessitates the application of highly concentrated and possibly hazardous CPAs (17–19). High-quality CPAs should possess excellent water solubility, membrane permeability, and little harmful effects at lower temperatures and higher concentrations (20, 21). Using higher concentrations of cryoprotectants, reducing solution volumes to lessen toxicity and osmotic stresses, and implementing innovative technologies like radiofrequency heating or nano-heating to fast and evenly rewarm are all likely to minimize freezing injury further (17, 22, 23). Cryopreservation by vitrification has effectively preserved heart valves, liver and kidney tissue sections, corneas, blood vessels, embryonic tiny structural tissues, and whole organs such as the liver and kidneys. In the cryopreservation of solid organs, the grafts were initially perfused with standard organ preservation solutions (e.g., UW solution) and subsequently stored at low temperatures (approximately 4℃) for preservation. Once the equipment was ready, the organs were connected to a specialized perfusion system, where they were first rinsed with a diluted carrier solution, with careful adjustments made to the pressure or flow rate. After attaining initial osmotic equilibrium, the organ was perfused with a full-strength carrier solution, supplemented with specialized nanoparticles, and subsequently placed in a controlled-rate freezer for vitrification. The vitrification techniques enabled the cryopreservation of rat kidneys for a duration of up to 100 days, with the ability for the gradual restoration of kidney function following nano-heated thawing and allografting (17). The same technique was applied to freeze and revive rat liver, successfully retaining the liver tissue architecture and vascular endothelial cells, enabling the liver to absorb indocyanine green and generate bile during reperfusion (19). The unsatisfactory results of vitrification-based cryopreservation for the heart may be attributed to the loss of myocardial tone caused by uneven freezing and rewarming, which inhibits the myocardium’s ability to pump blood. Inspired by the cryo-tolerance characteristics of the North American wood frog, the intracellular delivery of non-coding RNA not only activates the intrinsic antioxidant system but also enables cells to develop cold tolerance and reduce graft freezing damage (24). This technique optimizes the preservation of vascular endothelial cell integrity in the donor organs and mitigates IRI and immune rejection.

## 3 Subzero non-freezing preservation protocols

Considering the difficulties and risks linked to cryopreservation protocols, the subzero non-freezing preservation protocol, also known as the supercooling preservation protocol, has been explored concurrently. This technique enables the preservation of organs without freezing at subzero temperatures by employing organ preservation solutions enriched with high concentrations of osmotic cryoprotectants to perfuse and submerge the organs, regulating the height of the gas-liquid interface, while simultaneously monitoring and regulating the preservation temperature in real-time. A newly developed organ preservation system based on isochoric supercooling facilitates real-time monitoring of the preservation process, maintains the stability of the preservation solution, and prevents the formation of ice nuclei at subzero temperatures without the addition of CPAs, thereby enabling the extended preservation of large grafts (25). The subzero non-freezing preservation protocol has been validated in models of heart, liver, and kidney transplantation, extending organ preservation time by a minimum of 48 h (26, 27). Prevalent CPAs presently accessible include glycerol, methanol, ethanol, propanediol, ethylene glycol, butylene glycol, dimethyl sulfoxide, polyethylene glycol, 3-O-methyl-glucose, and antifreeze proteins (12, 26–28). Trehalose is a disaccharide commonly present in plants, animals, and microbes in nature. Rat lungs were preserved using ET-Kyoto solution with trehalose at 4°C and −2°C for 17 h, followed by perfusion with low-oxygenated blood. It was observed that lungs preserved at −2°C exhibited reduced arterial pressure, higher tidal volume and arterial partial pressure of oxygen, decreased endothelial cell damage, and increased intracellular adenosine triphosphate (ATP) levels (28). The superior protective effect may be associated with trehalose in the ET-Kyoto solution. A human liver transplantation model was used to verify the protective effect of the University of Wisconsin (UW) solution with glycerol and trehalose as adjuvants. This approach safely preserves the liver at −4°C without freezing for 33–42 h, retaining its viability after subnormothermic machine perfusion (SNMP) (29). It is important to emphasize that the livers in this experiment were not subjected to *in vivo* transplantation or later functional analyses. The use of 3-O-methyl-glucose and polyethylene glycol as CPAs prevented intra- and extracellular freezing at −6°C and maintained rat liver viability for 96 h. Following rewarming and homotransplantation, liver survival rates were 100 and 58% for 72 and 96 h of preservation, respectively. However, recipient rats exhibited delayed recovery of consciousness and movement throughout the initial postoperative week, with some experiencing weight loss (30). Porcine kidneys can be securely preserved at −5°C for 120 h with a peptoid-based preservation solution, which has demonstrated superior effectiveness compared to the UW solution for urogenesis, blood flow, and oxygen consumption following hypothermic machine perfusion (HMP) (31). The subzero non-freezing preservation protocol presents a promising alternative for organ preservation; nevertheless, these advantages have yet to be substantiated in large animal models. Moreover, the stability and safety of the subzero non-freezing preservation protocol are suboptimal, as random icing influenced by the volume of supercooled preservation solutions and the freezing characteristics following vibration could hinder the transport of donor organs. The toxicity of CPAs, osmotic stress, and IRI may also adversely impact the organ’s functional recovery.

## 4 Static cold storage protocols

SCS is now the standard practice for organ preservation, and the development of organ preservation solutions has been crucial in reducing graft injury during cold storage. The preservation process is straightforward, necessitating that the organ be submerged in the same solution at around 4℃ after being perfused with the organ preservation solution. For kidney preservation, Celsior solution, HTK solution, and UW solution are more favorable than Eurocollins solution in terms of the incidence of DGF (31). Currently, the most commonly used lung preservation solution is Perfadex solution, which contains glucose and the colloidal osmotic agent dextran and can prevent cell swelling and endothelial cell damage and reduce the incidence of primary graft dysfunction (32, 33). The selection between UW and HTK solutions for static liver preservation remains controversial. Despite the International Liver Transplantation Society’s disapproval of HTK solution for preserving donation after circulatory death (DCD) livers, Foley et al. utilized a comprehensive national database to compare the two preservation options, revealing that HTK solution exhibited an equivalent protective effect to UW solution in the preservation of DCD livers (34, 35). Given that various organs exhibit distinct tolerances to ischemia and hypothermia and that the composition of organ preservation solutions influences organ viability and clinical outcomes, it is essential to customize preservation strategies for individual organs. Table 1 summarizes the composition of widely used SCS solutions.

**Table 1:** Composition of current preservation solutions/overview.

Constituents (mmol/l)	UW	HTK	HTK-N	EC	Perfadex	CE	LPDG	IGL-1	IGL-2	TiProtec
Na^+^	29	15	16	15	138	100	138	125	125	16
K^+^	125	10	10	115	6	15	6	25	25	93
Ca^2+^		0.01	0.02		0.27	0.25		0.03	0.5	0.05
Mg^2+^	5	4	8		0.8	13	0.8	5.5	5	8
Cl^−^	20	50	30	15	142		142			103.1
Zn^2+^									0.091	
${\mathrm{SO}}_{4}^{2-}$	5				0.8		0.8	5	5	
${HCO}_{3}^{-}$				10						
HEPES									10	
Phosphate	25			58	0.8		0.8	25	25	1
Histidine		180	124			30			30	
Histidine.HCl		18								
N-acetyl-L-histidine			57							30
Allopurinol	1							1		
Glutathione	3					3		3	9	
Tryptophan		2	2							2
Aspartate			5							5
Adenosine	5							5	5	
Glutamic acid						20				
α-ketoglutarate	1	1	2							2
Alanine			5							5
Glycine			10							10
NaNO_2_ (nmol/L)									50	
L-arginine			3							
Raffinose	30							30		
Glucose				180	5		5			10
Saccharose			30				9.1			20
Mannitol		30				60		60	60	
polyethylene glycol 35								1	5	
Dextran 40					50 g/L		50 g/L			
Lactobionate	100					80		80	100	
HES (g/l)	59									
Deferoxamine			0.025							0.082
LK614			0.0075							0.017
Viscosity (cP)								1.2	1.7	
pH	7.4	7.2	7.0	7.2	7.4	7.3	7.4	7.4	7.4	7.0
Osmolarity (mOsm/L)	320	310	302	375	325	320	295	320	360	305

HEPES, 4-(2-Hydroxyethyl)piperazine-1-ethanesulfonic acid; HES, hydroxyethyl starch; CE, Celsior; LPDG, low potassium dextran glucose solution.

**Figure 1:**
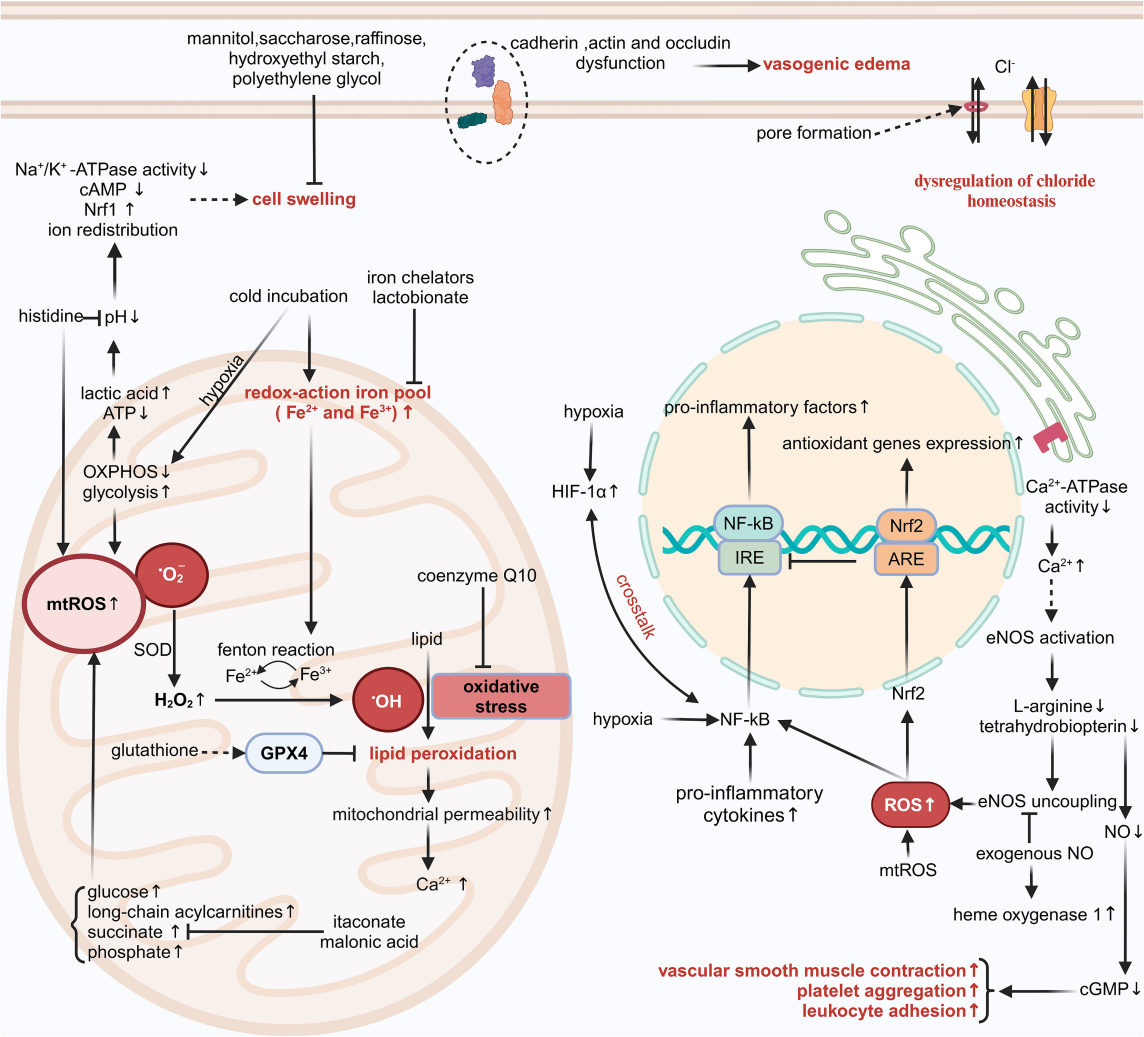
The injury mechanisms associated with SCS. cGMP, cyclic guanosine monophosphate; ARE, antioxidant responsive element; mtROS, mitochondrial ROS; Nrf2, nuclear factor-erythroid 2-related factor 2; cAMP, cyclic adenosine monophosphate; H_2_O_2_, hydrogen peroxide. Created in BioRender. Ran, Q. (2025) https://BioRender.com/z95k029, licensed under Academic License.

Donor organs are underutilized because of geographic limitations on donor-recipient matching and the restricted *in vitro* preservation duration. The application of organ preservation solutions at around 4°C for perfusion and immersion of grafts could inhibit cell metabolism and sustain organ viability for 4–6 h (36). Donor heart storage longer than 4 h is related to primary graft dysfunction (37). An optimal long-term survival rate was observed in pancreatic grafts with cold ischemia durations under 12 h. The incidence of graft failure increased by 1.2–1.4 times when pancreatic cold ischemia time ranged from 12 to 24 h (38). Previously, a duration of 6 h was considered the maximum permissible cold ischemia time for lung transplantation (39). Nonetheless, extensive distribution of donor lungs frequently requires the utilization of allografts with ischemia times exceeding 6 h. There is a dispute concerning the maximum duration of SCS of the lungs. Some reports indicate that a storage duration exceeding 6 or 8 h does not influence grade 3 primary graft dysfunction within 72 h after transplantation, reintubation, extracorporeal membrane oxygenation post-transplantation, acute rejection within 30 days, duration of hospital stay, and 5-year survival rates (39, 40). Unfortunately, the optimization of various existing organ preservation solutions cannot attain a significant breakthrough in SCS duration due to the inherent limitations of SCS technology. Contemporary biochemical and basic molecular biology techniques have facilitated a deeper comprehension of the injury mechanisms behind SCS (Figure 1).

### 4.1 Cold-induced injury

The SCS of grafts reduces ATP consumption and extends preservation, but the low temperature may induce additional injury (2). The cold-induced injury occurs independently of hypoxia/reoxygenation injury and is linked to increased chelatable iron pool and disruption of chloride (Cl^−^) homeostasis, ultimately resulting in apoptosis (41–44).

#### 4.1.1 Chelatable iron pool and Fenton reaction

The majority of intracellular iron is tightly linked to proteins, with just a tiny percentage (0.2–3%) constituting the chelatable iron pool (45). The intracellular pool of redox-active iron (Fe^2+^/Fe^3+^) increased swiftly following the initiation of cold incubation at 4°C; however, this augmentation was reversible upon rewarming after a brief cold incubation duration (2, 43, 45, 46). Cells incubated with HTK solution, histidine-lactobionate solution, and CE solution at 21°C and 37°C, respectively, still exhibited cytotoxicity. This damage was associated with significant lipid peroxidation, which could be mitigated by the antioxidants trolox, butylated hydroxytoluene, N-acetylcysteine, and membrane-permeable iron chelators such as 2,2′-dipyridyl, 1,10-phenanthroline, LK614 LK616, and deferoxamine. The testing of individual components of different organ preservation solutions, utilizing a modified Krebs–Henseleit buffer, indicated that the cytotoxicity of these solutions originates from histidine and phosphate (47, 48). The two substances are closely linked to the production of iron-dependent reactive oxygen species (ROS).

Oxidative stress refers to the imbalance between ROS and antioxidants within cells. Low-reactivity ROS, such as hydrogen peroxide, can interact with redox-active iron and generate highly reactive species, including iron-oxygen species and hydroxyl radicals, in a process referred to as the Fenton reaction (2). The hydroxyl radical induces various forms of damage, including lipid peroxidation, alterations in mitochondrial permeability, and a loss in mitochondrial membrane potential, particularly observed in hepatocytes and vascular endothelial cells (45, 46). Modified TiProtec solution and HTK-N solution contain the iron chelators desferrioxamine, which is hydrophilic and exhibits poor membrane permeability, and LK614, which is lipophilic and demonstrates good membrane permeability. Additionally, these solutions partially or completely substitute histidine with N-acetyl-L-histidine, effectively inhibiting the generation of hydroxyl radicals (4, 5, 49). Lactobionate in UW solution, CE solution, and Institut Georges Lopez-1 (IGL-1) solution also chelates ferric iron and mitigates oxidative damage during SCS (50). These organ preservation solutions also contain the antioxidant glutathione. Glutathione peroxidase 4 utilizes the tripeptide glutathione as a cofactor and is essential in mitigating lipid peroxidation (51). Coenzyme Q10 also functions as an effective antioxidant; however, its poor water solubility and stability present challenges for adding it into organ preservation solutions as a pharmaceutical agent.

#### 4.1.2 Dysregulation of chloride homeostasis

A decrease in serum Cl^−^ concentration will increase mortality risk in individuals with hypertension, heart failure, or myocardial infarction (52, 53). In recent years, there has been growing evidence that intra- and extracellular Cl^−^ concentrations and Cl^−^ channels are intimately related to IRI (54, 55). The decrease in intracellular Cl^−^ concentration can inhibit the formation of the nicotinamide adenine dinucleotide phosphate oxidase complex and promote the combination of vascular endothelial growth factor receptor 2 with protein tyrosine phosphatase 1B, thus preventing the activation of oxidase-mediated signaling of vascular endothelial growth factor receptor 2 and ultimately suppressing angiogenesis (56). In a recent multicenter, double-blind, randomized, controlled trial of DCD kidney transplantation, the administration of a balanced low-Cl^−^ crystalloid solution (Plasma-Lyte 148) in place of a 0.9% normal saline solution for the maintenance of blood volume was associated with a decreased incidence of dialysis treatment within 7 days post-transplantation (57). Consequently, the balanced crystalloid solution is recommended as the gold standard method of intravenous infusion for DCD kidney transplantation.

Maintaining appropriate intra- and extracellular Cl^−^ concentrations is crucial not only during the reperfusion process following transplantation but also during the SCS of grafts. A comparative analysis of cold incubation of hepatocytes utilizing modified Krebs–Henseleit buffer and various common organ preservation solutions indicated that cold-induced iron-nondependent injury stemmed from the higher Cl^−^ concentration in the preservation solutions; however, this damage was relatively mild (43). The incorporation of L-arginine into the HTK solution, the partial substitution of histidine with N-acetyl-L-histidine, and the reduction of Cl^−^ concentration led to a modified HTK fluid. This Cl^−^-deficient HTK solution improved myocardial systolic and diastolic force post-heart transplantation in rats; nevertheless, this advantageous effect could not be definitively ascribed to Cl^−^ deficiency (58). To clarify the impact of Cl^−^ concentration in preservation solutions on preservation effectiveness, Wu et al. conducted a comparison between a new HTK solution with low Cl^−^ concentration and one with high Cl^−^ concentration in a mouse heart transplantation model. Their findings indicated that the preservation solution with high Cl^−^ concentration significantly decreased myocardial injury and resulted in a higher graft survival rate, but this model cannot differentiate whether the benefits of HTK solution variants with high Cl^−^ concentration are due to positive effects on cardiac cells or endothelial cells (59). Comparable research protocols and outcomes of experiments have also been validated in a rat liver transplantation model, with Cl^−^ toxicity manifesting only when concentrations exceed 40 to 50 mmol/L (43, 60). Consequently, we reach a preliminary conclusion that the extent of iron-nondependent injury associated with SCS has a U-shaped correlation with the Cl^−^ concentration in the storage solution.

The mechanism by which extracellular Cl^−^ concentration induces cell death at low temperatures remains ambiguous, while alterations in intracellular pH and the activation of the early intrinsic apoptosis pathway might relate to this phenomenon. The ATP-gated P2X_7_ receptor is a plasma membrane receptor that is part of the P2X purinoceptor family. The P2X_7_ receptor agonist 2′-3′-O-(4-benzoylbenzoyl)-ATP can elicit membrane potential depolarization, pore formation, cell shrinkage, and lactate dehydrogenase release. Replacement of Cl^−^ in the culture medium with gluconate inhibits cell contraction and the release of lactate dehydrogenase mediated by the P2X_7_ receptor, suggesting that pore formation and extracellular Cl^−^ inflow are crucial in P2X_7_ receptor-induced apoptosis (61). Apoptosis is differentiated from necrosis by a characteristic volume loss or apoptotic volume decrease, which is attributed to ion redistribution. The UV-C light-induced changes in intracellular Cl^−^ concentration in Jurkat T cells influenced the activation of the mitogen-activated protein kinase signaling pathway at an early stage of the apoptotic signaling cascade. This apoptotic feature could be inhibited by the Cl^−^ channel inhibitor disodium 4-acetamido-4-isothiocyanato-stilbene-2,2-disulfonate (62). The Cl^−^ channels in the cell membrane exhibit a lack of specificity regarding the transport of Cl^−^. Alongside Cl^−^, these channels can also transport other anions such as halides, pseudohalides, and bicarbonates, exhibiting a higher transport efficiency than Cl^−^. Members of the dimorphic chloride intracellular channels (CLICs) family are extensively distributed across various intracellular compartments and exhibit unique characteristics, including a single transmembrane domain and a dimorphic existence as either soluble or membranous forms. They play a significant role in membrane trafficking, cytoskeletal function, apoptosis, cell cycle regulation, and vascular endothelial growth factor-mediated angiogenesis (63). CLIC1, CLIC4, and CLIC5 are abundant in rodent hearts, with CLIC4 localized to the mitochondrial outer membrane and CLIC5 found in the mitochondrial inner membrane (64). An acidic pH facilitates the conversion of CLICs into a membrane-associated conformation, enhancing the exposure of the hydrophobic inter-domain interface. Additionally, they found that purified CLIC5 can function as a fusogen, interacting with membranes to induce fusion, as evidenced by the increased diameter of liposomes and the mixing of lipids and contents between liposomes (65). Despite extensive research over several decades, definitive evidence supporting the function of CLICs as Cl^−^ channels remains absent, hindering a comprehensive understanding of their physiological role.

#### 4.1.3 Microvascular diastolic dysfunction

The coronary circulation resembles the renal circulation and exhibits strong auto-regulation capabilities (66–69). Microvascular resistance is crucial for blood perfusion within a specific microzone (70). Utilizing organ preservation solutions at 4–5°C for the perfusion and immersion of donor organs effectively lowers cell metabolism and prolongs storage duration. However, low-temperature liquid immersion significantly affects blood flow. The contraction of microvessels results in inadequate perfusion and supply of energy precursors in specific regions. Additionally, the buffering capacity against metabolic acidosis is compromised (71). The dysfunction of coronary vasodilatory functions is a significant, independent predictor of cardiac mortality in patients not having coronary artery disease (72). The activation of the sympathetic nervous system induced by cold exposure does not significantly affect blood flow in transplanted denervated human hearts, suggesting impaired vasodilatory functions of coronary arteries (73). Successive warm and cold ischemia also impairs the vasodilation of the DCD kidneys. Unfortunately, the vasodilatory functions cannot be improved by the constituents of the UW solution or IGL-1 solution (74). The rapid function deterioration of vascular smooth muscle cells may be intricately linked to the intimal hyperplasia observed in late graft failure.

Nitric oxide (NO) is a gaseous, lipophilic nitrogen oxide. The diminished NO bioavailability is a prevalent characteristic of endothelial dysfunction in cardiovascular disease. Low quantities of NO exhibit a significant anti-apoptotic effect in healthy organisms and can also mitigate cold-induced injury in isolated grafts (75). During SCS, the NO produced by endothelial nitric oxide synthase (eNOS) decreases with the consumption of L-arginine and tetrahydrobiopterin. Following reperfusion, most of the eNOS is suddenly activated by an influx of extracellular calcium. The deficiency of L-arginine and tetrahydrobiopterin results in increased superoxide generation by eNOS, further exacerbating oxidative stress. This procedure is known as the “uncoupling” of eNOS (76, 77). Prior research indicates that exogenous NO not only inhibits platelet aggregation and leukocyte adhesion and diminishes the inflammatory response, but also activates guanylate cyclase and enhances the synthesis of cyclic guanosine monophosphate, which induces myosin dephosphorylation in vascular smooth muscle and ultimately facilitates vascular smooth muscle relaxation and augments organ perfusion and energy reserves (78–80). Moreover, exogenous NO enhances both systolic and diastolic cardiac performance post-reperfusion, inhibits eNOS uncoupling, and increases the production of the antioxidant enzyme heme oxygenase-1, hence diminishing superoxide generation and apoptosis (75, 81). Some works of literature have reported the direct sources of NO or substrates of NO synthase, including nitroglycerin, S-nitroso human serum albumin, nitrosothiols, and L-arginine, as adjuvants in organ preservation solutions (82–85). These substances exhibit superior protective effects in improving the microvascular function of the liver, kidneys, and heart.

### 4.2 ATP depletion and accumulation of metabolic substrates

The limited ATP generated by anaerobic metabolism permits the grafts to survive just a brief duration of ischemia. A shortage of cellular ATP inhibits the activity of endoplasmic reticulum calcium-ATPase in renal tubular cells, hepatocytes, and cardiomyocytes, resulting in elevated cytosolic calcium levels. The opening of the mitochondrial membrane permeability transition pore during ischemia may subsequently result in mitochondrial calcium overload (2). Calcium-dependent proteases, including phospholipase A2 and calpain, are rendered inactive in acidic environments. Following reperfusion, the restoration of intra- and extracellular pH, along with mitochondrial calcium overload, activates these proteases, resulting in cell damage (86). The direct administration of ATP disodium into the coronary arteries during percutaneous coronary intervention has demonstrated enhancement of ventricular wall motion function, reduction of no-reflow rates, and mitigation of reperfusion injury (87, 88). Nonetheless, the poor membrane permeability of ATP challenges its direct application as an energy source for donor organs. The research employed adenosine, a metabolite of ATP, as its focal point. Adenosine is presently utilized as a substrate for the synthesis of ATP in UW, IGL-1, and Institut Georges Lopez-2 (IGL-2) solutions (89, 90). Aspartate can undergo transamination to produce oxaloacetate, which functions as a substrate in the tricarboxylic acid cycle and facilitates ATP production. Prior research indicates that a preservation solution supplemented with aspartate and α-ketoglutarate enables renal proximal tubules to generate ATP via anaerobic metabolism and sustain mitochondrial membrane potential, thereby mitigating hypoxia-reoxygenation mitochondrial injury (91). In recent years, modified HTK-N solutions have been developed by supplementing aspartate and α-ketoglutarate (already present in the HTK solution) to provide a tricarboxylic acid cycle substrate. This preservation solution ameliorates the deficiency of energy precursors during the SCS of grafts (82, 86, 92, 93). CE solutions utilize glutamate as a precursor for the synthesis of ATP (94). The addition of adequate energy precursors to organ preservation solutions not only creates a modest quantity of ATP but also moderately slows the accumulation of intermediates from the tricarboxylic acid cycle.

Increased mitochondrial ROS production can frequently be observed in diseases characterized by mitochondrial dysfunction, and an important hallmark of these diseases is reduced or impaired oxidative phosphorylation and enhanced glycolysis enzyme activity, indicating that mitochondrial ROS production is closely linked to glycolysis processes (5, 95). ROS-induced glucose uptake can, in turn, influence ROS generation and elimination. Elevated extracellular glucose concentrations can promote spontaneous glucose auto-oxidation and the synthesis of advanced glycation end products, along with collateral glucose metabolism, which involves hexosamine, protein kinase C, and polyol pathways (96). These processes collaborate to cause increased oxidative stress and damage to cell structures. When the delicate balance between glucose-induced ROS formation and the intrinsic antioxidant system as well as glucose-mediated ROS scavenging is disrupted, intracellular ROS and glucose levels will increase in a vicious cycle, ultimately resulting in cell death. Long-chain acylcarnitines are intermediates of detrimental long-chain fatty acids. In ischemic myocardium, the activity of carnitine palmitoyltransferase 1 is elevated while that of carnitine palmitoyltransferase 2 is diminished, resulting in the progressive accumulation of long-chain acylcarnitines within the intermembrane space of mitochondria. This accumulation causes hyperpolarization of mitochondrial membrane potential, inhibition of oxidative phosphorylation, and excessive generation of ROS (97).

The accumulation of succinate in mitochondria is a prevalent metabolic characteristic of ischemic tissues. The synergistic effect of fumarate overflow resulting from purine nucleotide decomposition and the partial reversal of the malate/aspartate shuttle induces a reversal of succinate dehydrogenase, finally resulting in the accumulation of mitochondrial succinate (98). Following reperfusion, succinate accumulated in the mitochondria is swiftly re-oxidized by succinate dehydrogenase; meanwhile, reverse electron transport through mitochondrial complex I facilitates the rapid and substantial production of ROS. The ROS pulse operates together with a series of damaging events following reperfusion, ultimately resulting in cell death (99). Malonic acid serves as a competitive inhibitor of succinate dehydrogenase. Dimethyl malonate, a prodrug of malonic acid, can mitigate IRI when administered before and during ischemia (99). Itaconate, a metabolite of *cis*-aconitate that accumulates concurrently with succinate during ischemia, can also function as a competitive inhibitor of succinate dehydrogenase. It serves as a regulator to modulate mitochondrial redox metabolism, inhibiting succinate accumulation and suppressing ROS generation (100–102). Experiments on large animals such as rabbits, dogs, and pigs have demonstrated that lungs preserved at 10°C have less mitochondrial damage and can withstand cold ischemia for a longer time. This protective effect may be due to the fact that lung tissue produces more itaconate at 10°C (100, 101, 103). So far, lungs preserved at 10°C have achieved a total clinical preservation time of up to 24 h without negative effects on organ function or short-term outcomes following transplantation. As a result, the appropriate temperature for donor lung preservation has been re-evaluated, and 10°C may become the standard criteria (104). Interestingly, most HMP is performed at temperatures ranging from 6 to 10°C, and this higher temperature may be another explanation for its superiority compared to SCS (4°C). The immune response gene 1 (IRG1) encodes an enzyme responsible for the production of itaconate. The histone deacetylase inhibitor valproic acid can induce the transcription of IRG1, which in turn promotes the nuclear translocation of nuclear factor erythroid 2-related factor 2 and the synthesis of heme oxygenase-1 as well as superoxide dismutase 1 in mouse cardiomyocytes. This process improves the utilization of itaconate and diminishes the accumulation of succinate during cold storage (37). The IRG1/itaconate pathway similarly activates nuclear factor erythroid 2-related factor 2-mediated antioxidant responses in hepatocytes and attenuates hepatic IRI (102). Targeting metabolic pathways to modulate metabolite concentrations is an attractive therapeutic option during SCS.

### 4.3 Hypoxia-induced cell swelling

Cell damage resulting from hypoxia and edema is both progressive and cumulative, with hypoxia-induced cell swelling inflicting greater damage than either condition independently. The extent of swelling correlates with the duration of SCS and the precise composition of the organ preservation solution selected (105). Under hypothermic hypoxic conditions, cell swelling is attributed to altered intra- and extracellular ion concentrations (driven by the Na^+^/H^+^ exchanger, Na^+^/ ${HCO}_{3}^{-}$ symporter, and Na^+^-K^+^-2Cl^−^ cotransporter), reduced levels of cyclic adenosine monophosphate, decreased Na^+^/K^+^-ATPase activity, and upregulated expression of nuclear respiratory factor 1, ultimately resulting in inadequate organ perfusion (86, 106–108). Pathological pore formation in cell membranes under hypoxic conditions can also facilitate the permeation of Na^+^ and Cl^−^, resulting in cell swelling, and this process can be prevented by glycine and alanine in HTK-N solution (3, 61). Moreover, the disruption of endothelial cell occludin, VE-cadherin, and F-actin results in increased capillary permeability, which subsequently promotes plasma leakage following reperfusion and induces vasogenic edema (106, 109). This implies that alterations in tight junctions and adherens junctions may be the fundamental cause of vasogenic edema linked to SCS. Injury to vascular endothelial cells predisposes the graft to acute rejection, resulting in primary graft dysfunction.

Maintaining moderate crystalloid or colloid osmotic pressure of organ preservation solutions helps to reduce microcirculatory disturbances. Mannitol has been mixed into HTK, CE, IGL-1, and IGL-2 solutions to alleviate hypoxia-induced cell swelling; nonetheless, mannitol can permeate the hepatocyte membrane, resulting in hepatocyte edema (110). Due to the inability of sucrose molecules to traverse cell membranes, the modified HTK-N solution utilizes sucrose in place of mannitol within the HTK solution (111). UW solutions utilize the relatively high molecular weight of anions, raffinose, lactobionate, and hydroxyethyl starch to alleviate SCS-induced cell swelling, demonstrating superior efficacy in reducing cardiac and pancreatic endothelial cell injury. However, certain studies indicate that high-viscosity hydroxyethyl starch could trigger acute kidney injury and elevate mortality risk (50, 112, 113). Although sucrose and raffinose cause cell shrinkage, moderate cell shrinkage seemingly does not impact cell function as well as cell membrane integrity (110). Low potassium dextran glucose solution uses dextran to sustain osmotic pressure in the solution and demonstrates superiority in lung preservation (114). Polyethylene glycol 35 is a non-immunogenic, non-toxic, water-soluble polyether compound that improves the activity of the vasodilator NO and the mitochondrial enzyme aldehyde dehydrogenase 2, regulates immunogenicity and ameliorates microcirculatory abnormalities following SCS and reperfusion (115, 116). It serves as a colloidal osmotic agent for IGL-1 and IGL-2 solutions, presenting superior protection for fatty liver grafts during SCS and oxygenated hypothermic machine perfusion (HMPox) (90). Clarysse et al. have demonstrated that polyethylene glycol 35 can stabilize the endothelial glycocalyx in a rat intestinal IRI model, consequently restricting reperfusion-mediated cell swelling, bacterial translocation, and inflammatory responses (117). Hyperbranched polyglycerol, a compact branched polymer characterized by low viscosity, is a promising colloid for the preservation of donor organs. In a mouse cardiac allograft transplantation model, hyperbranched polyglycerol decreased neutrophil infiltration and markedly improved cardiac function and tissue integrity (118).

### 4.4 Inflammation response

Hypoxia is believed to be the fundamental cause of inflammation and immunological response (119). Hypoxia can also interact with the inflammatory response, causing further deterioration of organ function and, in particular, promoting the advancement of inflammatory diseases (120). A common hallmark of perioperative organ failure in surgical patients is a hypoxia-induced inflammatory response, suggesting that hypoxia itself is an inflammatory stimulation (121, 122). Hypoxia and inflammation are related to the activation of the hypoxia-inducible factor (HIF) and the nuclear factor κB (NF-κB) transcription factor family, respectively. HIF and NF-κB co-regulate the effector functions of immune cells and increase susceptibility to hypoxia (123, 124). HIF is crucial for the activation and increased phagocytosis of macrophages and neutrophils, while NF-kB is involved in the proliferation of several immune cells, including T cells, B cells, and dendritic cells (125). The expression of the endotoxin receptor Toll-like receptor 4 and the conduction of NF-κB-mediated inflammatory signaling during kidney transplantation increased progressively with the prolongation of SCS. Research utilizing Toll-like receptor 4 knockout mice observed diminished renal IRI, implying that prolonged ischemia and hypoxia of donor organs amplify inflammatory responses through the Toll-like receptor 4 signaling pathway (126). Active inflammation also causes tissue hypoxia, which results from the concentration of inflammatory cells (including neutrophils, eosinophils, and monocytes), higher rates of cellular oxidative metabolism, and increased activity of various oxygen-consuming enzymes (119, 121).

There is extensive crosstalk between the two molecular signaling pathways, HIF and NF-κB, as they share a number of activating stimulus sources, modulators, and targets (120, 125). Hypoxia has been demonstrated to stimulate the co-activation of the HIF and NF-κB signaling pathways in a manner dependent on IκB kinase and transforming growth factor β-activated kinase 1 (127, 128). Elevated NF-κB activity stimulated by inflammatory cytokines promotes the transcription of messenger RNA of HIF-1α, and the negative feedback of HIF-1α can also regulate NF-κB transcriptional activity under inflammatory conditions (129, 130). Signaling mediators, including S-2-hydroxyglutarate, hydrogen sulfide, and ROS, activated by tissue inflammation, also play a role in the regulation of HIF activity in immune cells (131). There is also reciprocal feedback between oxidative stress and the inflammatory response, which leads to further damage of grafts. ROS are powerful inflammatory initiators that promote the synthesis and release of many pro-inflammatory mediators, including prostaglandin E2, leukotriene B4, tumor necrosis factor-α, interleukin-1β (IL-1β), and IL-6 (132, 133). The metabolite itaconate, which accumulates during SCS, not only inhibits succinate accumulation but also exhibits anti-inflammatory properties. The knockdown of IRG1 intensified injury to grafts and systemic inflammatory responses, while the metabolic reprogramming of itaconate by upregulating the expression of IRG1, which encodes the enzyme responsible for itaconate production, improved cardiac and hepatic function after extended SCS (37, 38, 102). Targeting immunometabolic pathways could be a promising method for improving graft outcomes.

### 4.5 Ischemia–reperfusion injury

Prolonged ischemia results in irreversible organ damage; however, tissue damage persists and exacerbates following reperfusion. This phenomenon is known as IRI (134). Prior research indicates that IRI encompasses multiple pathological mechanisms, including apoptosis, necrosis, ferroptosis, calcium overload, oxidative stress, no-reflow phenomenon, inflammatory response, protease activation, subcellular remodeling, cell hypertrophy, extracellular matrix remodeling, and fibrosis, among others (51, 135–137). The mechanisms of IRI partially overlap with those previously discussed and will not be described in detail here. It is crucial to stress that the ischemia typically described in these publications is warm ischemia. The cascade pathways of injury associated with prolonged cold ischemia of grafts partially differ from those of warm ischemia; for instance, in porcine DCD kidneys, warm ischemia preferentially activates caspase-1, while cold ischemia significantly elevates caspase-3 activity and triggers tubular apoptosis (219). Multiple studies have documented the application of caspase inhibitors aimed at the apoptotic pathway as a prospective strategy to enhance graft performance. While caspase inhibition mitigated the severity of post-transplantation injury, it failed to avert cellular damage entirely (220–222). This indicates a simultaneous activation of alternative damage pathways. In recent years, controlled reperfusion approaches, also known as ischemic postconditioning, which regulate blood pressure, acid-base balance, oxygen saturation, and ion concentrations during reperfusion, continue to be investigated to alleviate reperfusion injury (223). However, each variable section in the ischemia-reperfusion process further complicates these damage mechanisms. During hibernation, animals decrease their basal metabolic rate, and comprehending the mechanisms of organ protection during this state may elucidate the pathophysiology of DGF. However, animal organs do not experience true ischemia during hibernation, which more accurately parallels the HMPox protocol (224, 225). Additional advancements in the protective benefits of SCS seem challenging to attain. Ischemia and reperfusion are two consecutive processes, and IRI inevitably occurs when grafts are preserved by SCS, thus making dynamic MP an attractive field of research.

## 5 Machine perfusion

The requirement to improve post-transplantation survival of ECD (which includes DCD donors) grafts and recent advances in MP technology have resulted in the development of clinically effective *ex vivo* MP devices for liver, heart, lung, and kidney grafts that maintain organ viability for up to 24 h. MP entails linking the organ’s own blood vessels to a perfusion system that continuously supplies the isolated organ with a perfusate containing oxygen, nutrients, and therapeutic agents during the preservation and transportation phase. Long-term *ex vivo* dynamic MP not only maintains microvascular tone, enhances aerobic metabolism, and excretes toxic metabolites, but also repairs ECD grafts and evaluates graft viability (8, 9). The MP also aids in diminishing the occurrence of DGF, even when utilized to preserve grafts from standard criteria donors (138). Currently, MP technology encompasses multiple aspects like perfusion temperature, oxygen saturation, selection of perfusion solution, continuous versus pulsatile perfusion, duration of perfusion, evaluation of organ viability, and organ repair. Owing to the varied types of grafts and their functional conditions, a standardized MP technique is presently unavailable.

### 5.1 Temperature of machine perfusion

The efficiency of cell energy metabolism correlates directly with ambient temperature. Based on the perfusion temperature, the MP strategies are categorized into HMP, SNMP, controlled oxygenation rewarming, and normothermic machine perfusion (NMP). The nonoxygenated HMP (0–8°C) protocol initially perfuses the graft directly using an existing organ preservation solution. This approach enables the organ to achieve a lower metabolism, sustain intra- and extracellular pH levels, and mitigate cell swelling (1). The advantages and disadvantages of HMP protocols and SCS protocols for the preservation of DCD kidneys have been controversial. The latest data from six organ procurement facilities in the United States indicate that the SCS protocol is less effective than the nonoxygenated HMP protocol in reducing the DGF of DBD kidneys (139). Oxygenation of the perfusate promotes intracellular ATP synthesis and delays ischemic injury, which is known as the HMPox protocol (1, 140, 141). Multicenter clinical controlled trials have demonstrated that HMPox-preserved livers have a lower risk of primary nonfunction and nonanastomotic biliary strictures compared to conventional SCS protocols (142, 143). The SNMP protocol (12–34°C) is currently in an awkward situation. Although this protocol not only reduces cell energy metabolism but also allows for organ repair/recovery, experimental animal studies have shown mixed results. In an *in vitro* preservation experiment of DCD kidneys, the SNMP protocol markedly increased blood flow, urine output, and creatinine clearance and maintained renal structural integrity in comparison to HMPox and SCS. Nonetheless, in this research, the preservation effect of SNMP was not compared with NMP. In fact, the NMP protocol appears to be more advantageous than SNTM for kidney preservation (144). Research on liver transplantation indicates that fatty livers produce more ATP during SNMP compared to NMP; however, glutathione is significantly depleted in hepatocytes during SNMP, which is probably attributed to the hepatocytes’ inability to overcome the threshold energy necessary for glutathione synthesis under subnormothermic conditions (145). Controlled oxygenation rewarming (8–20°C) is the relay process of SCS and HMP, enabling a slow and precisely controlled rewarming of grafts to prevent mitochondrial malfunction and the activation of apoptotic signaling pathways resulting from excessively rapid rewarming (146). NMP (35–38°C) adopts a membrane oxygenator to oxygenate erythrocytes or other oxygen carriers within the perfusate. An adequate supply of oxygen and nutrients can restore the grafts to a physiological metabolic state, allowing the functional assessment and repair of ECD grafts (8, 147, 148). This approach effectively prevents cell injury resulting from hypothermia and the rewarming process, offering substantial benefits in suppressing vasospasm, safeguarding vascular endothelial cells, and suppressing immune rejection (7, 149). NMP can restore ATP depleted during ischemia in isolated porcine kidneys, enabling metabolic recovery, and is capable of maintaining the equilibrium of acid–base and the integrity of renal tubular structure compared to SCS, HMP, and SNMP (144, 150). Several studies have demonstrated that normothermic conditions (about 36°C) are the optimal temperature for the preservation of liver grafts. NMP can lower lactate levels during hepatic perfusion *in vitro*, maintain hepatic metabolic activity, and lower the risk of early allograft dysfunction (151, 152). As a result, the NMP technique has been gradually applied to clinical liver transplantation.

### 5.2 Perfusate composition and oxygen carriers

The composition of the perfusate is essential for grafts during in vitro MP preservation, similar to SCS. The ideal perfusate should include oxygen carriers, suitable concentrations of diverse ions or colloidal osmotic agents, acid–base buffering agents, energy precursors, and growth factors (8). Currently, perfusates are mainly classified as banked blood (BB), plasma-free red cell-based solutions, and acellular perfusates. The oxygenation of the crystalloid or colloid organ preservation solution, commonly applied in clinical practice, supplies dissolved oxygen that may only satisfy the metabolic requirements of the grafts during HMP and SNMP at temperatures below 20°C (141). So far, BB has been successfully used as a perfusate for the extended preservation of the lungs, liver, kidneys, and heart (153–155). Nevertheless, conflicting evidence persists about the application of BB in cardiac NMP. The TransMedics OCS Heart advocates for using BB to perfuse the heart; however, clinical trials conducted by Chew et al. indicate that BB is inappropriate as a perfusate for donor hearts (156). The intricacies of BB and the potential hazards of immunological responses, thrombosis, hemolysis, and infectious disease transmission have resulted in the progressive substitution of blood products with novel perfusates featuring diverse, precisely defined compositions (157). The plasma-free red cell-based solutions comprise physiological oxygen carriers and establish an anti-inflammatory milieu free of platelets and leukocytes, presenting advantages over BB (149). Prolonged NMP following the oxygenation of a plasma-free red cell-based solution could progressively rehabilitate the impaired function in marginal liver grafts. However, when the perfusion duration surpasses 24 h, the iron in hemoglobin will gradually oxidize, resulting in an accumulation of methemoglobin (158). The incorporation of the antioxidant N-acetylcysteine into plasma-free red cell-based solutions can efficiently inhibit the synthesis and accumulation of methemoglobin (159). Furthermore, the oxygenator applied during extended MP can trigger platelet dysfunction and damage the integrity of red blood cells, resulting in increased hemolysis in the perfusate (158). Comparatively, membrane oxygenators are more effective than other types of oxygenators in reducing hemolysis.

Considering the hemolysis of erythrocytes, a variety of artificial oxygen carriers have been manufactured (Table 2). These oxygen carriers exhibit superior oxygen-transport capabilities; nonetheless, certain kinds have been documented to possess nephrotoxic, ophthalmotoxic, or induce systemic vasoconstriction (160). Erythrocruorin, a giant metalloprotein in invertebrates like annelids and crustaceans, is comparatively safe. It not only facilitates effective oxygen delivery but also possesses anti-inflammatory qualities (161). However, there is currently no human red blood cell substitute acknowledged by the United States Food and Drug Administration. The search for a suitable oxygen carrier remains an appealing research direction since it would significantly reduce the waste of BB and allow for mass production of perfusates, finally resulting in cost savings.

**Table 2:** Overview of the advantages and disadvantages of different artificial oxygen carriers in machine perfusates.

Machine perfusates	Oxygen carriers	Advantages	Disadvantages	References
Banked whole blood	Red blood cells	Low production of methemoglobin; Close to physiological conditions	Waste of BB; Immune responses; Hemolysis; Thrombus formation; Blood-borne infectious transmission; Cross-matching difficulties; Rapid increase in blood potassium; Blood lactate increased; High viscosity	Chew et al. (156) and Laing et al. (157)
Plasma-free red cell-based solutions	Red blood cells	Low production of methemoglobin; Creates an anti-inflammatory environment without platelets and leukocytes; Physiological oxygen carrier	Waste of BB; Immune responses; Hemolysis; Biochemical and humoral variations; Blood-borne infectious transmission; Cross-matching difficulties	Hosgood and Nicholson (149) and Laing et al. (157)
Acellular perfusates	Water	Provides sufficient oxygen at low temperatures; Direct use of preservation solutions for SCS	Not suitable for SNMP and NMP; Requires oxygenation under high PO_2_ conditions; Amino acid oxidation	Panisello Rosello et al. (115), Doorschodt et al. (161), Minor et al. (185), Iwata et al. (186), and Minor et al. (187)
	Hemoglobin	No cross-matching required; Long-term storage; Low liquid viscosity; Natural oxygen carrier	Production of methemoglobin; Vasoconstriction; Short half-life; Nephrotoxicity; Removal of nitric oxide	Zin et al. (188), Jansman et al. (189), and Jahr et al. (190)
	Perfluorocarbon	High O_2_ affinity; Obey Henry’s law; Can be mass-produced	Risk of vision loss; Requires oxygenation under high PO_2_ conditions; Instabilities of PFC emulsions; Transient drop in neutrophils and platelets; Flulike symptoms; Hypertension; Pulmonary complications	Jägers et al. (191) and Ye et al. (192)
	Iron(II) porphyrin	Covalently binds to serum albumin without changing its structure and function; No cytotoxicity	Difficult to synthesize; Easily binds to endogenous carbon monoxide	Kitagishi et al. (193) and Wang et al. (194)
	Liposome microbubbles	Molecular imaging; Targeted drug/gene delivery; Easily diffuse across membranes; Low drug bioaccumulation	Short circulatory lifetime (<1 h); Relatively large (micron) sizes; Repetitive administration increases serum viscosity	Sirsi et al. (195), Tao and Ghoroghchian (196), and Fix et al. (197)
	Erythrocruorin	Simple gradient release O_2_; High O_2_ affinity; Non-immunogenic; Potential anti-inflammatory, anti-bacterial, and antioxidant properties; Slight vasodilation; Good stability; Long circulating half-life; Low toxicity	Only used in preclinical HMP and clinically in SCS	Kruczkowska et al. (198)
	Nanomaterials	Can be mass-produced	Low water solubility; Easy to aggregate into large particles; Hepatorenal toxicity; Short half-life	Rubeo et al. (199, 200)

### 5.3 Organ function evaluation

The viability evaluation and screening of grafts before transplantation can decrease the risk of adverse events post-transplantation. The MP parameters and the analysis of perfusate composition are frequently employed to evaluate graft activity; nevertheless, these data typically exhibit constrained prediction accuracy. The MP parameters frequently employ hemodynamic data as a reference, including vascular resistance (the ratio of perfusion pressure to blood flow) and hemodynamic alterations following the administration of vasoactive drugs (162–165). Functional parameters obtained by left ventricular catheterization offer an effective assessment of myocardial performance during MP of cardiac grafts (165). A study published over a decade ago provided a comprehensive review of contrast-enhanced ultrasound, three-dimensional ultrasound, and magnetic resonance spectrometry techniques for assessing renal perfusion and ATP levels preoperatively (166, 167). The resonance Raman spectroscopy technique is a novel, real-time, non-invasive method for assessing graft function which uses a 441 nm laser and high-resolution spectroscopy to quantify the oxidative status of mitochondrial cytochromes during perfusion. This approach elucidates mitochondrial function and the degree of graft damage, enabling the customization of optimal reoxygenation strategies (168). Evaluating the extent of mitochondrial damage in DCD livers during HMP by analyzing flavin mononucleotide and nicotinamide adenine dinucleotide levels in the perfusates through mass spectrometry and fluorimetry can forecast liver function (169). Indocyanine green fluorescence imaging during HMP of liver grafts may also provide valuable insights into the functional evaluation and selection of marginal livers following the damage-repair process (170). These non-invasive techniques provide rapid access to test results in a sterile operating environment and present benefits over conventional assessment methods.

Components of the perfusate applicable for evaluating graft injury include organ damage markers (such as cardiac enzymes), activated immune cells, lipid peroxidation products, lactate, electrolytes, oxygen partial pressure of perfusate, and both anti-inflammatory and pro-inflammatory cytokines (171, 172). The concentration of bilirubin, creatinine, and urea nitrogen in the perfusate, together with urine and bile output, are critical parameters for evaluating the vitality of renal tubular cells and hepatocytes. Variations in pH, bicarbonate, and glucose levels between MP perfusate and bile have also been demonstrated to be reliable indicators of bile duct cell functionality (165, 173). The extracellular vesicles originating from the donor grafts can be released into the blood and urine, which could reflect the graft’s functional condition. The analysis of nanoparticles, including extracellular vesicles, in perfusate and urine during NMP can evaluate renal quality before and during transplantation (174, 175). Cell-free microRNAs serve as sensitive biomarkers for early tissue damage. The quantitative reverse transcriptase polymerase chain reaction analysis of microRNAs within perfusate and bile indicated that the quantities of hepatocyte-derived microRNA-122 and cholangiocyte-derived microRNA-222 could indicate bile secretion function and the severity of cholangiocyte injury after 6 h of NMP (176, 177). Future research may focus on transcriptomic analysis of inflammation-related gene expression during MP to evaluate graft function.

### 5.4 Marginal organ repair

Graft viability is closely linked to the occurrence of primary nonfunction and DGF following transplantation (172). In comparison with grafts from DBD and living donation, ECD grafts suffer more severe IRI and exhibit a higher incidence of post-transplant acute rejection, graft insufficiency, or graft loss, hence adversely impacting the long-term survival of the grafts (178–180). Although HMPox can increase ATP levels and decrease mitochondrial ROS formation during reperfusion, the practical application of this technique for organ repair remains debatable (1, 145, 181). The warm, oxygen- and nutrient-rich perfusate sustains the graft in a near-physiological metabolic condition, enabling both the evaluation of graft function and the targeted administration of therapeutic agents in an isolated environment, thereby offering a chance for functional repair of marginal organs and cell regeneration. This comparatively isolated environment not only blocks inflammatory cell infiltration and immunological rejection but also mitigates the adverse consequences of systemic administration of drugs. Prolonging the *ex-vivo* dynamic MP preservation of grafts beyond 24 h could theoretically facilitate marginal organ repair and cell regeneration while eliminating metabolically detrimental waste products; however, this depends on the further development of more technologically sophisticated perfusion equipment (182, 183). Prevalent techniques for graft repair encompass anti-inflammatory therapy, stem cell therapy, RNA interference therapy, and nutrient delivery (9, 184) (Table 3). These techniques could improve organ function and augment graft survival via multiple mechanisms. The successful salvage of marginal organs helps mitigate the imbalance between the supply and demand for grafts.

**Table 3:** Strategies of graft repair during MP.

Repair strategies	Materials	Outcomes	Animal models	References
Anti-inflammatory therapies	MCC950	↓NLRP3 inflammasome pathway activation; ↓IL-1β; ↓hepatocytes apoptosis	Pig kidney HMP allograft model	Yu et al. (201)
	Emricasan	↓Liver fibrosis; ↓alanine minotransferase	Human liver transplantation model	Weinberg et al. (202)
	PFCOC	↓Myeloperoxidase activity; ↓potassium and lactate; ↓vascular resistance; ↑ATP	Rat *ex vivo* lung SNMP model	Arni et al. (203)
	Hemoadsorber	↓Gene transcription of IL-1B, NLRP3, caspase1 and neutrophil recruitment chemokines	Human kidney NMP model	Ferdinand et al. (204)
Gene therapies	C5 siRNA-LNP	↓Expression and activity of complement C5; ↑graft survival	Rat kidney transplantation model	Ishigooka et al. (205)
	Lentiviral vectors	↑IL-10, MIP-1α, MIP-2, IP-10, epidermal growth factor; ↓IL-12, IL-17, MCP-1, IFN-γ	Rat kidney transplantation model	Yuzefovych et al. (206)
Therapeutic gasses	Carbon monoxide	↑Cyclic guanosine monophosphate, heme oxygenase-1; ↑p38 phosphorylation; ↓JNK phosphorylation; ↓IL-6 and IL-1b mRNA	Rat lung transplantation model	Dong et al. (207)
	NO	↓Vascular resistance	Pig kidney pulsatile MP model	Gage et al. (208)
	Hydrogen sulfide	↓Oxygen consumption; ↓mitochondrial activity; unchanged ATP levels	Porcine kidney NMP model	Maassen et al. (209)
Cellular therapies	Mesenchymal stromal cells	Mesenchymal stromal cells were retained in the renal cortex and did not affect plasma creatinine, glomerular filtration rate, or neutrophil gelatinase-associated lipocalin concentrations	Porcine kidney NMP and auto-transplantation model	Lohmann et al. (210)
	Multipotent adult progenitor cells	295 unique proteins with immunomodulatory potential were found in the perfusate	Human liver NMP model	Laing et al. (211)
	hAECs	↑IL-6, IL-10, and G-CSF; ↑gene expression of IL-6, IL-10, HLA-G, HLA-E, and PDL-1	Rat-isolated pancreatic islets model	Lebreton et al. (212)
	T regulatory cells	↓Donor-specific antibodies in recipient mice; preserved graft vascular structure; ↑graft survival	Murine cardiac allograft model	Masaoka et al. (213)
	Extracellular vesicles	↑COX IV-1; ↑HGF and VEGF; ↓caspase-3; improved renal ultrastructure	Human kidney HMPox model	Rampino et al. (214)
Nutritional support	Citric acid	Kidney metabolism was maintained in an active state for up to 4 days.	Human kidney NMP model	De Haan et al. (215)
Remove metabolic waste	Continuous renal replacement therapy	Livers were successfully preserved in a perfusion system with physiological perfusate for 100 h.	Human liver NMP model	Nalesso et al. (216)
Antioxidant therapy	Alb-NC	↑Glutathione levels; ↑SOD and catalase activity; ↓mitochondrial DNA4977 deletion	Human liver NMP model	Del Turco et al. (217)
	N-alkyl-O-HTCC	↑SOD2 and ALDH2 activity in mitochondrion	Rabbit kidney HMP model	Zhang et al. (218)

NLRP3, NOD-like receptor protein 3; PFCOC, perfluorocarbon-based oxygen carriers; C5 siRNA-LNP, lipid nanoparticle formulation of small-interfering RNA against complement C5; MIP-1α, macrophage inflammatory protein-1alpha; IP-10, interferon-γ-induced protein 10; MCP-1, Monocyte chemotactic protein-1; hAECs, human amniotic epithelial cells; G-CSF, granulocyte colony-stimulating factor; HLA-G, human leukocyte antigen G; COX, cytochrome c oxidase; HGF, hepatocyte growth factor; JNK, c-Jun N-terminal kinase; VEGF, vascular endothelial growth factor; PDL-1, programmed death ligand 1; Alb-NC, nanoceria conjugated with albumin; N-alkyl-O-HTCC, N-alkylated-O-(2-hydroxyl) propyl-3-trimethyl ammonium chitosan chloride; SOD, superoxide dismutase; ALDH2, mitochondrial acetaldehyde dehydrogenase 2.

## 6 Conclusion and outlook

Organ preservation solutions are one of the most significant instruments in organ transplantation, and lowering cell metabolism or minimizing the graft’s ischemia duration *in vitro* is a prerequisite for maintaining graft viability. Cryopreservation, subzero non-freezing preservation, and SCS adopt low temperatures to diminish cell metabolism, hence improving graft ischemic tolerance. With the growing demand for organ transplants, doctors are compelled to use grafts from ECD donors; nonetheless, these grafts are more susceptible to harm, and conventional static preservation techniques might further worsen the grafts. The elimination or partial substitution of potentially toxic substances in organ preservation solutions serves as an important modification strategy for these solutions. In other words, the high levels of hazardous CPAs will inevitably restrict the clinical application of cryopreservation and subzero non-freezing preservation protocols. Despite the costly expense and technical complexity of long-term *ex vivo* dynamic MP, which increases the discard of grafts in the event of preservation failure, it offers multiple benefits over conventional static storage methods, particularly in enabling the dynamic assessment of organ viability and facilitating organ repair and regeneration. The successful repair and regeneration of grafts relies mainly on the prolonged duration of *ex vivo* MP as well as the development and utilization of safe and effective artificial oxygen carriers and therapeutic agents. Interestingly, several energy precursors utilized in organ preservation solutions for SCS, together with therapeutic medicines documented in the literature, are probably compatible for direct incorporation into machine perfusates. The spiral development of organ preservation strategies necessitates reliance on established research findings and the emergence of innovative technologies. Consequently, artificial oxygen carriers and organ repair/regeneration techniques require further investigation. This review offers a systematic overview of advancements in diverse organ preservation techniques, which we believe will contribute to the optimization and innovation of organ preservation strategies.
